# Pharmacological and Behavioral Characterization of D-473, an Orally Active Triple Reuptake Inhibitor Targeting Dopamine, Serotonin and Norepinephrine Transporters

**DOI:** 10.1371/journal.pone.0113420

**Published:** 2014-11-26

**Authors:** Aloke K. Dutta, Soumava Santra, Horrick Sharma, Chandrashekhar Voshavar, Liping Xu, Omar Mabrouk, Tamara Antonio, Maarten E. A. Reith

**Affiliations:** 1 Wayne State University, Department of Pharmaceutical Sciences, Detroit, MI, United States; 2 University of Michigan, Department of Pharmacology and Chemistry, Ann Arbor, MI, United States; 3 New York University, Department of Psychiatry, New York, NY, United States; 4 New York University, Department of Biochemistry and Molecular Pharmacology, New York, NY, United States; Nathan Kline Institute for Psychiatric Research and New York School of Medicine, United States of America

## Abstract

Major depressive disorder (MDD) is a debilitating disease affecting a wide cross section of people around the world. The current therapy for depression is less than adequate and there is a considerable unmet need for more efficacious treatment. Dopamine has been shown to play a significant role in depression including production of anhedonia which has been one of the untreated symptoms in MDD. It has been hypothesized that drugs acting at all three monoamine transporters including dopamine transporter should provide more efficacious antidepressants activity. This has led to the development of triple reuptake inhibitor D-473 which is a novel pyran based molecule and interacts with all three monoamine transporters. The monoamine uptake inhibition activity in the cloned human transporters expressed in HEK-293 cells (70.4, 9.18 and 39.7 for DAT, SERT and NET, respectively) indicates a serotonin preferring triple reuptake inhibition profile for this drug. The drug D-473 exhibited good brain penetration and produced efficacious activity in rat forced swim test under oral administration. The optimal efficacy dose did not produce any locomotor activation. Microdialysis experiment demonstrated that systemic administration of D-473 elevated extracellular level of the three monoamines DA, 5-HT, and NE efficaciously in the dorsal lateral striatum (DLS) and the medial prefrontal cortex (mPFC) area, indicating in vivo blockade of all three monoamine transporters by D-473. Thus, the current biological data from D-473 indicate potent antidepressant activity of the molecule.

## Introduction

Major depressive disorder is a debilitating illness affecting 15–20% of the population in the United States [1]. According to the World Health Organization by 2020 it would be the second-most leading cause of disability worldwide making it a global health problem. It is believed that 20% of all individuals suffer from a major mood disorder at least once in their lifetime. Depression is potentially fatal since most sufferers consider life threatening acts and suicide [2], [3].

The underlying causes of depression are still unclear and 15–20% of depressed patients are resistant to all known therapies. Unipolar depression is ranked as number one before all other somatic and psychiatric illnesses. Main stream therapy for depression involves drugs which are selective serotonin reuptake inhibitors (SSRIs) and serotonin/norepinephrine reuptake inhibitors (SNRIs). However, there still remains a significant unmet need for much more improved therapy, as large numbers of depressed people, an estimated 15–30%, are still refractory to the current existing therapies. Thus, current therapy is less than ideal with remission rates of only 25–35% and response rates of 45–60% [4]. Besides these, slow onset of action of the current therapies along with other associated side effects indicate an unmet need for better therapy for treatment of MDD. In the current pharmacotherapy of depression, a dopaminergic component has not been included in spite of existence of evidences pointing to a strong dopaminergic component in depression [5]. The medial prefrontal cortex brain area has been shown to be associated with depressed mood and sadness and neuroimaging studies indicated certain deficiencies of neuronal activities in this area with depressed subjects [6]. This region receives innervation from all three monoamines, thus, restoration in the imbalanced level of monoamines by antidepressants has been shown to improve symptoms of depression [7]. Dopamine has been linked to depression for some time [5], [8], [9], [10]. Since dopamine controls mood and emotion, reduced dopaminergic activity leads to decreased motivation, production of anhedonia and loss of interest. A dysfunctional dopaminergic system in the mesocorticolimbic pathway may lead to development of anhedonia associated with loss of pleasure and interest along with loss of motivation [5]. An antidepressant capable of increasing dopamine should address these symptoms.

**Figure 1:**
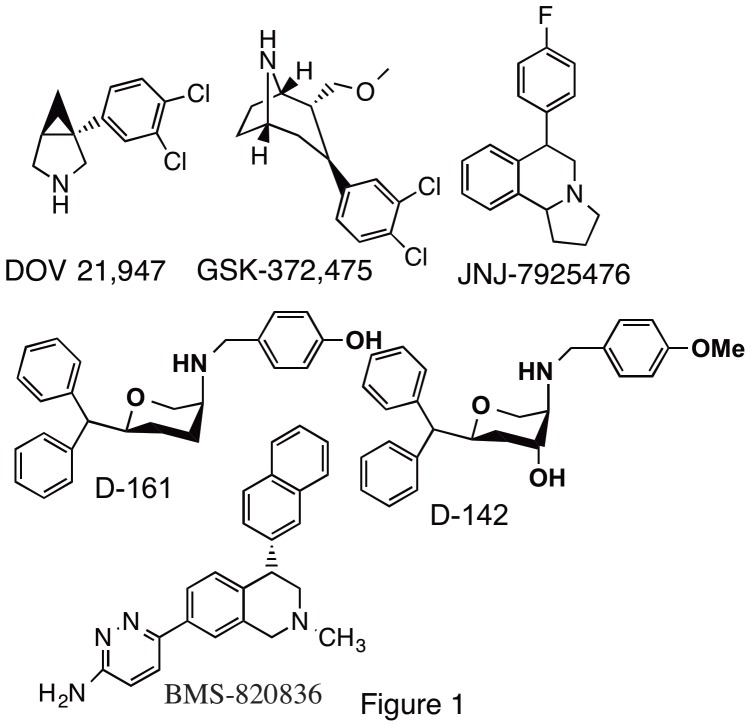
Molecular structures Triple reuptake inhibitors.

Triple monoamine reuptake inhibitors (TRIs) have recently been advanced as agents that can exert potent antidepressant activity with potentially a lower side-effect profile [1], [11]. The underlying involvement of dopaminergic system in depression prompted our efforts to develop triple reuptake inhibitors, which are expected to produce strong antidepressant effects in addition to the treatment of anhedonia which is prevalent in MDD. A successful adjunct therapy approach involving the combination of the dopamine transporter blocker bupropion and an SSRI was found to be more efficacious in patients refractory to SSRI [9], [10]. In this regard, it is important to mention that there are only few compounds which are known to exhibit a DNRI-type profile. One well known example of this is bupropion, used as an antidepressant agent in the clinic [9], [12]. In recent years triple reuptake inhibitors (TRIs) inhibiting all three monoamine transporters have been hypothesized to produce greater efficacy due to additional dopamine activity [1], [13], [14], [15]. A number of TRIs, e.g. DOV 21,947, PRC200-SS, JNJ-7925476, BMS-820836 and GSK-372,475 have been developed (Figure 1) and have been characterized in animal depression models [16], [17]. Recently, DOV 21,947 has undergone Phase 2b/3a clinical trial which demonstrated promising efficacy in a treatment-resistant MDD patient group. Moreover, both DOV 21,947 and BMS-820836 indicated high level of safety and tolerability in human [18], [19]. The concern related to dopamine activity in TRIs leading to addiction was alleviated in a recent study indicating that the racemic version of DOV 21,947 has much less addictive-like properties compared to cocaine [20].

In our TRI development effort, we have initiated the design and synthesis of novel asymmetric trisubstituted and disubstituted pyran derivatives as inhibitors of monoamine transporter systems in the CNS. CNS-active pyran compounds are relatively rare. Interestingly, a number of molecules from this pyran series exhibited a TRI profile of potent interaction with dopamine, serotonin and norepinephrine transporters [21], [22], [23], [24], [25], [26]. Compounds with other profiles, including SNRIs and DNRIs were also identified. A number of lead TRIs, specifically D-142 and D-161 (Figure 1), were shown to be efficacious in the animal models of depression [21], [26], [27], [28]. The results also indicated blood brain barrier crossing ability of these compounds. One of our lead TRIs, identified as D-473, displayed potent uptake inhibition at all three monoamine transporters in both rat tissues and in the cells expressing cloned human transporters. D-473 exhibited oral activity as well as excellent in vivo efficacy in animal models for depression under oral administration conditions, indicating oral bioavailability with good brain penetration and site specific interaction in the CNS.

## Materials and Methods

### Reagents and drugs

**Figure 2:**
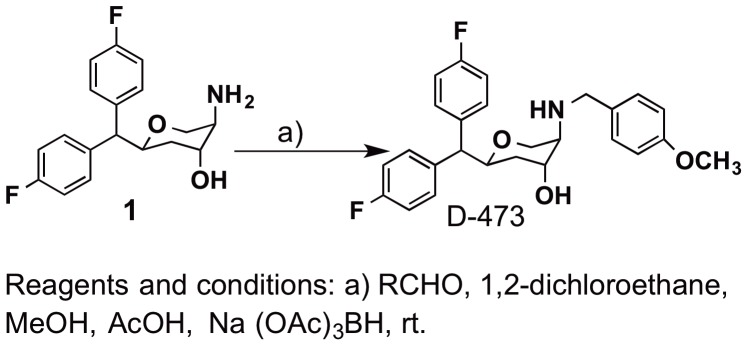
Synthesis of D-473.

Synthesis of D-473 ((2*S*,4*R*,5*R*)-2-(bis(4-fluorophenyl)methyl)-5-((4-methoxybenzyl)amino)tetrahydro-2*H*-pyran-4-ol) is shown in Figure 2 and is described below. This compound was synthesized by asymmetric synthetic pathway as developed by us [25]. [Ring 2,5,6-^3^H]dopamine (38.7 Ci/mmol), [1,2-^3^H]serotonin (28.0 Ci/mmol), and levo-[ring-2,5,6-^3^H]norepinephrine (44.6 Ci/mmol) were obtained from Perkin-Elmer (Boston, MA, U.S.A). Imipramine, desipramine, fluoxetine, and reboxetine, and GBR 12909 dihydrochloride (1-[2-[bis(4-fluorophenyl)methoxy]ethyl]-4-[3-phenylpropyl]piperazine) were purchased from SIGMA-ALDRICH (St. Louis, MO). All reagents used to perform microdialysis (i.e. for aCSF) and saline solutions, as well as LC-MS reagents were purchased from Sigma Chemical Co (St Louis, MO).

### Animals

Male Sprague-Dawley rats (200–225 g) were purchased from Harlan. Animals were housed in a temperature and humidity controlled room with 12 h light/dark cycle. Food and water were accessible to animals freely through out the duration of study. All testing occurred during the light component. All animal procedures were reviewed and approved by Wayne State University animal investigation committee consistent with AALAC guidelines. Microdialysis experiments performed at the University of Michigan were treated as approved by the University of Michigan Unit for Laboratory Animal Medicine (ULAM) and in accordance with the National Institute of Health (NIH) Guidelines for the Care and Use of Laboratory Animals.

### Chemistry

#### Synthesis of (2S,4R,5R)-2-(bis(4-fluorophenyl)methyl)-5-((4-methoxybenzyl)-amino)-tetrahydro-2H-pyran-4-ol (D-473)

Amine **1** (200 mg, 0.63 mmol) was reacted with 4-methoxybenzaldehyde (90 mg, 0.66 mmol), glacial acetic acid (25 µL, 0.41 mmol), and Na(OAc)_3_BH (199 mg, 0.93 mmol) in a mixture of 1,2-dichloroethane (6 mL) and methanol (2 mL). The residue was purified by gradient silica gel column chromatography using a mixture of dichloromethane and methanol (100∶1 to 6∶1) to afford corresponding compound **D-473** (200 mg, 73%) as a white solid (Figure 2). ^1^H NMR (400 MHz, CDCl_3_): δ 7.06–7.39 (m, 6 H), 6.94 (dd, *J* = 16.7, 8.5 Hz, 4 H), 6.84 (d, *J* = 7.9 Hz, 2 H), 4.39 (t, *J* = 9.4 Hz, 1 H), 4.04 (s, 1 H), 3.59–4.0 (m, 8 H), 2.62 (s, 1 H), 1.68 (t, *J* = 11.43 Hz, 1 H), 1.41 (d, *J* = 14.1 Hz, 1 H).

^13^C NMR (100 MHz, CDCl_3_): δ 162.7, 162.6, 160.3, 160.2, 159.4, 137.5, 137.4, 137.3, 137.2, 129.9, 129.8, 129.7, 129.6, 128.3, 115.6, 115.4, 115.3, 115.1, 114.2, 73.8, 65.6, 63.6, 56.3, 55.3, 54.6, 50.3, 32.9.

[α]^25^_D_ = (−) 48.7°, *c* = 1 in MeOH. The product was converted into corresponding hydrochloride salt, m.p.: 190–205°C. Anal. Calcd for [C_26_H_27_F_2_NO_3_.HCl.H_2_O] C, H, N.

The product was converted into corresponding mesylate salt, m.p.: 200–205°C. Anal. Calcd for [C_26_H_27_F_2_NO_3_.CH_3_SO_3_H] C, H, N.

#### Inhibition of monoamine uptake by cloned human biogenic amine transporters in heterologous cells

Inhibition of substrate uptake by cloned human transporters was measured with stably transfected human embryonic kidney (HEK) 293 cells as in our previous work [29]. The cell lines were obtained and used in uptake assays as described in the same paper [29]. [^3^H]DA ([ring 2,5,6-^3^H]dopamine (45.0 Ci/mmol, Perkin-Elmer, Boston, MA, U.S.A) was used for monitoring DAT and NET (please note that DA is an excellent substrate for NET, for our previous discussion see Santra et al., 2012). [^3^H]5-HT ([1,2-^3^H]serotonin (27.9 Ci/mmol, Perkin-Elmer) was the radioligand for monitoring SERT.

Drug stocks contained an additional 0.01% (w/v) bovine serum albumin in order to reduce absorption of drug to the walls of the assay plates. At least five triplicate concentrations of each test compound were studied, spaced evenly around the IC_50_ value which was converted to *K*_i_ with the Cheng-Prusoff equation. [21] With observed K_m_ values and applied [^3^H]ligand concentrations, the conversion factors (multipliers applied to IC_50_ for calculating K_i_) were>0.84 (Table 1).

**Table 1:** Inhibition of Drugs at hDAT, hSERT, and hNET in HEK293 Cells as Determined in Uptake Assays.

Drugs	DAT uptake, Ki, nM, [^3^H]DA a	SERT uptake, Ki, nM, [^3^H]5-HT^a^	NET uptake, Ki, nM [^3^H]DA^a^
D-142	179±27	5.43±1.40	21.2±4.3
**D-473**	**70.4±16.7**	**9.18±1.69**	**39.7±7.6**
Fluoxetine	5,126±3,260	14.4+1.1	1,755±444
Desipramine	4,051±1,029	57.3±10.2	0.86±.04
Citalopram		3.66±0.64	
Benztropine	90±16^b^		
GBR 12909	22.3±4.4	780±195	261±112

a Uptake into cells expressing hDAT, hSERT, or hNET was measured as described in Methods. Where indicated (superscript b), values are from our previous publication. Results are average±S.E.M. for 3 to 10 independent determinations.

b Chen et al., 2004.

#### Broad Receptor screening

Compound D-473 was characterized in several CNS receptor binding assays to assess selective and specific interactions in the CNS. The assays were carried out generously by the National Institute of Mental Health’s Psychoactive Drug Screening Program (NIMH PDSP). The NIMH PDSP is directed by Bryan L. Roth MD, PhD at the University of North Carolina at Chapel Hill and Project Officer Jamie Driscol at NIMH, Bethesda MD, USA (http://pdsp.med.unc.edu/).

Compound D-473 was first evaluated in primary binding assays targeting, among others, cloned human dopamine receptor subtypes, serotonin receptor subtypes, α-adrenergic receptors, and opioid receptors. The description of all receptors targeted and corresponding radioligand used, is provided in Table 2 in the Discussion section. The default concentration for primary binding experiments was 10 µM. Compound with inhibition>50% at 10 µM in the primary assay was moved into the secondary assay with full concentration curves of the test compound in order to calculate the Ki value for inhibition. For experimental details please refer to the PDSP web site <http://pdsp.med.unc.edu/> and click on “Binding Assay” or “Functional Assay” on the menu bar. We have also added a brief description of assay protocols in the supplementary material section.

**Table 2:** Binding affinity of D-473 for CNS receptors.

Target Receptor	Radioligand	% Inhibition of Binding at 10 µM D-473	Ki for D-473(nM)
D_1_	[^3^H]SCH 23390	62.2	630
D_2_	[^3^H]N-methylspiperone	42.9	
D_3_	[^3^H]N-methylspiperone	30.8	
D_4_	[^3^H]N-methylspiperone	37.2	
D_5_	[^3^H]SCH 23390	74.7	1571
5HT_1a_	[^3^H]8-OH-DPAT	35.4	
5HT_1b_	[^3^H]GR-125743	14.2	
5HT_1e_	[^3^H]5-HT	38	
5HT_1d_	[^3^H]GR-125743	39	
5HT_2a_	[^3^H]ketanserin	16.3	
5HT_2b_	[^3^H]LSD	70.8	597
5HT_2c_	[^3^H]Mesulergine	45.4	
5HT_3_	[^3^H] LY 278,584	3.5	
5HT_5a_	[^3^H]LSD	19.5	
5HT_6_	[^3^H]LSD	−11.4	
5HT_7_	[^3^H]LSD	41.4	
GABA _A_	[^3^H]Muscimol	5.1	
Alpha_1A_	[^3^H]Prazosin	23.6	
Alpha_1B_	[^3^H]Prazosin	16.6	
Alpha_1D_	[^3^H]Prazosin	41.8	
Alpha_2A_	[^3^H]Clonidine	14.5	
Alpha_2B_	[^3^H]Clonidine	25.4	
Alpha_2C_	[^3^H]Clonidine	61	>10,000
Beta_1_	[^125^I]Iodopindolol	24.6	
Beta_2_	[^125^I]Iodopindolol	−4.4	
Beta_3_	[^125^I]Iodopindolol	61.2	>10,000
BZP Rat Brain Site	^3^H-Flunitrazepam	−3	
δ-opioid	[^3^H]DADLE	42.7	
κ-opioid	[^3^H]Bremazocine	48.5	
H_1_	[^3^H]Pyrilamine	2.8	
H_2_	[^3^H]Tiotodine	70.1	141
H_3_	[^3^H]Alpha-methyl Histamine	−14.7	
µ-Opioid	[^3^H]Diprenorphine	20.5	
M_1_	[^3^H]QNB	15	
M_2_	[^3^H]QNB	−9.5	
M3	[3H]QNB	6.9	
M4	[3H]QNB	−16.7	
M5	[3H]QNB	3.2	
PBR		8.4	

The default concentration of drugs for primary binding experiments was 10 µM, and % inhibition (mean of 4 determinations) is shown. Except where indicated assays were with heterologous expression systems. Study was carried out by the National Institute of Mental Health’s Psychoactive Drug Screening Program.

#### Forced swim test in rats after oral administration

The experiment was carried out in the same way as described in our previous publications according to the Porsolt protocol [28], [30]. The subjects were male Sprague Dawley rats (Harlam Sprague Dawley Inc., Indianapolis, Ind., USA) weighing 200–225 g housed in cages for at least 1 week prior to testing. Animals were maintained in a temperature-controlled environment under a 12 hr light-dark cycle. All subjects were naive and were used only once.

Rats were transported to the testing room at least for one hour prior to testing for acclimatization and adaptation purposes. Experimental sessions were conducted between 9 AM to 2 PM daily. Animals were assigned randomly and were placed individually in a glass cylinder (24.5 cm×35.5 cm) filled with water at room temperature to a depth of 22 cm. All the test sessions were recorded by video cameras. The water was changed in the beginning of each session and the temperature was maintained constant at 24–25°C. Rats were judged to be immobile if they made only minimal movement, barely keeping afloat.

The test procedure consisted of a pretest and test session separated by 24 h [30]. During the pretest period, rats were placed in the swim chamber for 15 min. Followed by the initial swim exposure; rats were patted dry and were transferred to the individual cages. Drugs or vehicle were then orally administered (p.o) 15 min after the initial swim exposure and were then transported to their home cages. On the following day the rats were brought back to the testing room at least 1 h before the beginning of test session. Rats were administered either drugs or vehicle 1 h before the swim test. Each rat underwent a 5-min swim session, which was videotaped and scored later. In the case of imipramine, pretreatment time was 30 min.

Drug solutions were prepared freshly on the test days. Compound **D-473** was dissolved in 3% beta-hydroxy propyl cyclodextrin solution. All drug and vehicle preparations were administered orally. **D-473** was administered at a dose of 5, 10, 25 and 50 mg/kg and the volume was maintained at 1.2 mL/rat. Drug or vehicle was administered 1 h prior to testing for forced swimming. An individual, blinded to the treatment, scored the videotapes for immobility. Immobility scores were analyzed by one way ANOVA test.

#### Locomotor Activity

Sprague Dawley Rats were tested at 25 mg/kg oral dose of D-473 to monitor changes in any locomotor activity in acrylic Auto-Track/Opto-Varimex-4 System (Columbus Instrument; Columbus, Ohio). The purpose was to evaluate locomotor activity of the same doses of drug that were used in the forced swim test. Rats were acclimated in the test chambers for 1 h prior to oral administration of D-473. Locomotor activity of the drug was measured for a total of two hour post administration of the drug which corresponded to the time of measurement in the forced swimming experiment. GBR 12909 was studied as a control drug known to stimulate and was administered i.p. as in published studies; vehicle was orally administered.

#### Plasma and Brain uptake concentration of D-473

Sample preparation**:** Following oral administration of 25 mg/kg of **D-473** and **D-142** in male Sprague Dawley rats, the blood samples were collected at different time points over a period of 8 h for analysis. Concentrations of **D-473** and **D-142** in plasma were quantitated using a set of calibration standards prepared in blank matrix that were processed in parallel. Plasma concentration evaluation was carried out by the CRO, Absorption System, PA. Additionally, brain uptake studies of **D-473** were carried out at three different time points (2, 4 and 6 h) following oral administration of 25 mg/kg **D-473**. Concentrations of **D-473** in rat brain were quantitated using a set of calibration standards which were prepared in blank brain homogenate matrix on the day of analysis. The standard samples (5, 10, 25, 100, 500, 1000, and 2000 ng/mL) for calibration curve were prepared by spiking 45 µL of blank rat plasma with 5 µL of appropriate working dilution of **D-473** in acetonitrile. The bioanalytical sample preparation for brain and plasma analysis followed by quantification of test compounds entailed the addition of 250 µL acetonitrile to every 50 µL of standard and analyte samples to precipitate tissue plasma proteins and tissue macromolecules. These mixtures were then vortexed for 15 min at 1400 rpm. The suspensions were then clarified by centrifugation (18000 g, 10 min at 4°C), and each 150 µL of the resulting supernatant was mixed well with 50 µL 0.1% formic acid in 50/50 (v/v) ACN/water solution for about 10 sec. The mixtures were again centrifuged (18000 g, 10 min at 4°C) before 100 µL of clear supernatant were transferred to auto sampler vials for LC-MS/MS analysis.

#### Chromatographic analysis

The analysis of brain samples was performed using Waters (Milford, MA, USA) Acquity UPLC instrument with a triple quadrupole (TQD) MS analyzer. The LC-MS/MS detection was performed using a positive Multiple Reaction Monitoring (MRM) method by monitoring the ion transition of D-473 from *m/z* 440.39 → 121.03. The MS/MS conditions include capillary voltage, 2.92 kV, cone voltage, 26.0 V; source temperature, 150°C; desolvation temperature, 350°C; desolvation gas flow, 500 L/hr; and collision energy, 33 eV. The MS control and data acquisition were collected using the Waters MassLynx software v4.1. Chromatographic conditions were achieved using a reverse phase C-18 ethylene bridged hybrid column (BEH C18) (2.1×100 mm, 1.7 µM). 5 µL of sample solutions were injected and samples were eluted using 0.1% formic acid in water (solvent A) and 0.1% formic acid in ACN (solvent B) mixture with a flow rate of 0.2 mL/min. Gradient elution of the mobile phase was 90% A to 5% A from 0 to 1 min, 5% A, 95% B from 1 to 3 min, and 5% A to 90% A from 3 to 5 min.

The analysis of blood sample was carried out by the CRO Absorption system, PA. Briefly, the analysis was carried out by Waters Acquity UPLC equipped with PE Sciex API4000 mass spectrometer. Reverse phase Waters Acquity BEH Phenyl Column 1.7micron, 2.1×30 mm column was used for analysis. 5 µL of sample solutions were injected with a flow rate 1 mL/min and the samples were eluted with gradient elution using solvent A (10% buffer: 90% water) and solvent B (10% buffer: 90% acetonitrile). Buffer used was 40 mM Ammonium Formate Buffer, pH 3.5. Pharmacokinetic prarameters were estimated by a non-compartmental model using WinNonlin v5.2.1 software.

#### Microdialysis procedure and measurement of monoamines

Adult male Sprague-Dawley rats (Harlan, Indianapolis, IN) weighing between 250 and 350 g were used for all experiments. Rats were housed in a temperature and humidity controlled room with 12 hr light/dark cycles with food and water available *ad libitum*. Adequate measures were taken to prevent animal pain and discomfort. All animals were treated as approved by the University of Michigan Unit for Laboratory Animal Medicine (ULAM) and in accordance with the National Institute of Health (NIH) Guidelines for the Care and Use of Laboratory Animals.

Rats were anesthetized with using an isoflurane vaporizer that was interfaced to a model 963 Ultra Precise stereotaxic instrument with 0.1 mm resolution (David Kopf Instruments, Tujunga, CA). Concentrically designed microdialysis probes (1 mm dialyzing length) were built in house then implanted bilaterally into the dorsal lateral striatum (DLS) and the medial prefrontal cortex (mPFC) according to the following coordinates from bregma and dura (in mm): AP +1, ML +3.3, DV –5.7 and AP +3, ML −0.5, DV −5, respectively (Paxinos and Watson, 2007) [31]. Placement of microdialysis probes into the DLS and mPRC is shown in Figure S1. Each animal had a probe in both DLS and the contralateral mPFC. 1 mm probes were selected for higher spatial resolution and lower tissue damage compared to larger probes. Probes were secured to the skull by acrylic dental cement and metallic screws. Following surgery, rats were allowed to recover and experiments were run 24 hr after probe implantation. Microdialysis probes were flushed at a flow rate of 2 µl/min with aCSF (composition in mM: CaCl_2_ 1.2; KCl 2.7, NaCl 148 and MgCl_2_ 0.85) for 2 hr using a Chemyx Fusion 400 syringe pump (Chemyx, Stafford, TX). Perfusion flow rate was then reduced to 1 µl/min and samples were collected every 10 min. Baseline values were collected for 50 min and then the drug D-473 was injected (10 mg/kg, i.p.) and samples were collected for an additional 2 h. When experiments were completed, animals were euthanized and then brains were extracted and frozen at −80°C until histology.

#### Neurochemical analysis using LC-MS

Dopamine, norepinephrine (NE), serotonin (5HT), DOPAC, homovanillic acid (HVA) and 5-hydroxyindole acetic acid (5-HIAA) were analyzed using a benozyolation derivitization LC-MS method recently described by our laboratory [32]. Briefly, 10 µl dialysate samples were derivatized by adding 5 µl of 100 mM sodium tetraborate, 5 µl of 2% benzoyl chloride in acetonitrile, and then 5 µl of a stable ^13^C benzoylated isotope internal standard mixture was then added to improve quantitation. A Thermo Fisher Accela UHPLC (Waltham, MA) system automatically injected 5 µl of the sample onto a Waters (Milford, MA) HSS T3 reverse phase HPLC column (1 mm X 100 mm, 1.8 µm). Mobile phase A consisted of 10 mM ammonium formate and 0.15% formic acid. Mobile phase B was pure acetonitrile. Analytes were detected by an Thermo Fisher TSQ Quantum Ultra triple quadrupole mass spectrometer operating in multiple reaction monitoring (MRM) mode. The assay limits of detection were (nM in dialysate) for DA, 0.3; NE, 0.2; 5HT, 0.1; HVA, 0.5; DOPAC, 2; and 5HIAA, 5. Basal levels in dialysates were (nM) in mPFC: DA, 0.475; NE, 1.59; 5HT, 0.11; HVA, 103; DOPAC, 63; and 5HIAA, 328; and in DLS: DA, 7.74; NE, 1.09; 5HT, 0.10; HVA, 1055; DOPAC, 1655; and 5HIAA, 455. Average standard deviation in above basal levels was 18%.

#### Data presentation and statistical analysis

Behavioral results were analyzed with Prism 6.0 (GraphPad Software, San Diego, CA). The immobility time for forced swimming test was analyzed by one-way ANOVA (analysis of variance) followed by post Dunnett’s multiple comparison test vs. vehicle. Data for vehicle was used as negative control, respectively. Statistical significance was assumed in analysis if p≤0.05.

All neurochemical data were transformed into percent baseline with baseline values all averaging 100%. Statistical analysis was performed by t-test analysis of every point relative to baseline values (i.e. 100%) using Prism 6 (Graphpad Software Inc, La Jolla, CA). P values <0.05 were considered to be statistically significant.

## Results

### Inhibition of monoamine uptake into cells heterologously expressing hDAT, hSERT, or hNET

**Figure 3:**
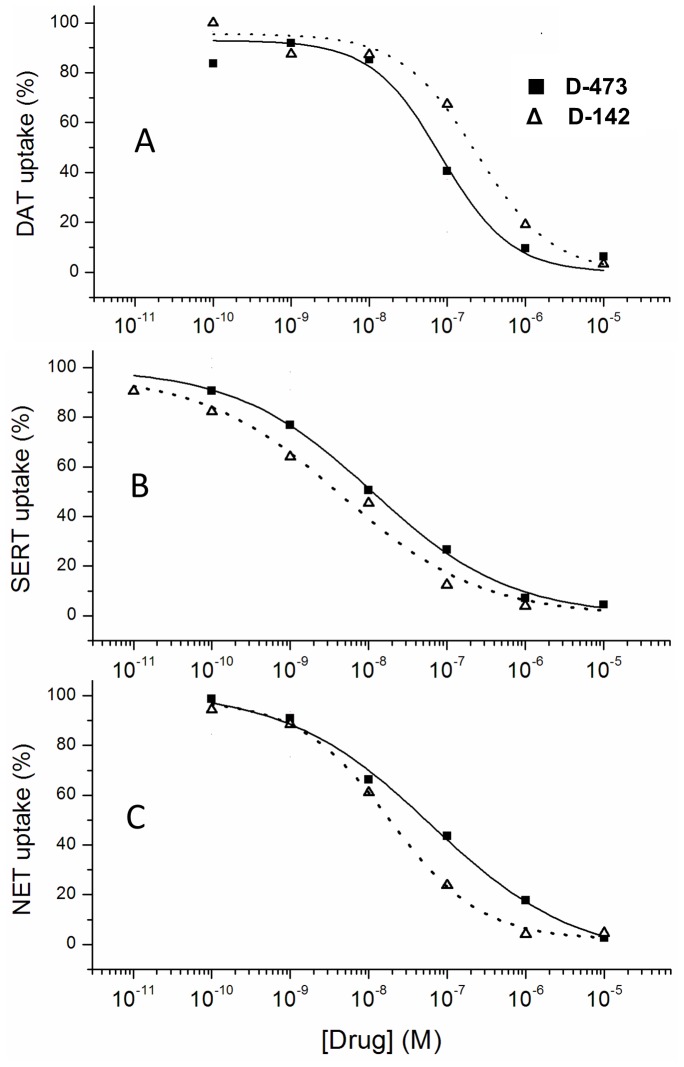
Inhibition of [^3^H]monoamine uptake by D-473 and D-142 in cells heterologously expressing hDAT, hSERT, or hNET (▪-, D-473; Δ-, D-142). A) Monoamine uptake by DAT, B) Monoamine uptake by SERT, and C) Monoamine uptake by NET was assessed as described in Methods and plotted according to the Logistics equation in the Origin fitting software (see Reith et al., 2012). Points shown are those obtained in a representative experiment, performed in triplicate, which was replicated 8–16 times.

D-473 was characterized in cells expressing cloned human transporters (Table 1, Figure 3). The compound showed the highest potency in inhibiting uptake by SERT, followed by NET and DAT. The potency at SERT was almost one and a half fold greater than that Fluoxetine, a well-known SSRI. The activity at NET was roughly two fold more potent than at DAT (39 nM vs 70 nM for NET and DAT, respectively). The standard reference compounds e.g. Desipramine (for NET), Citalopram (for SERT), Benztropine (for DAT) and GBR 12909 (for DAT) were assayed under the same conditions. It is important to note the potent DAT inhibitor GBR 12909 exhibited more than three-fold greater DAT inhibitory potency than D-473. For comparison purposes, the non-fluorinated version of the lead compound D-473, known as D-142, another triple uptake inhibitor, is also included in the present transporter assays (Table 1, Figure 3).

### Determination of specific interaction of D-473 in CNS broad receptors screening assays

Interaction of D-473 with various CNS GPCR receptors and ion channels was examined with various radioligands as shown in Table 2. Binding assays for dopamine and serotonin receptor subtypes did not indicate appreciable affinity of D-473 for these receptors (generally, Ki>10 µM; for D1, D5, and 5HT2b, Ki was>590 nM). Similarly, D-473 did not show any appreciable affinity for any alpha adrenergic or beta adrenergic receptors subtypes as shown in Table 2. D-473 also did not show any affinity for any of the opioid receptors, calcium channel and GABA receptor. Lack of any affinity for muscarinic receptor subtypes indicates that D-473 should not produce any muscarinic side effect such as dry mouth or constipation. Among all receptors tested, the histamine H2 receptor exhibited moderate affinity for D-473 (141 nM). Overall, the data from Table 2 indicate that D-473, beyond interacting with the three monoamine transporters, DAT, SERT and NET in the CNS, does not display appreciable affinity for brain targets as assesses in a wide receptorome ligand binding screen.

### In vivo data from forced swim test (oral administration)

**Figure 4:**
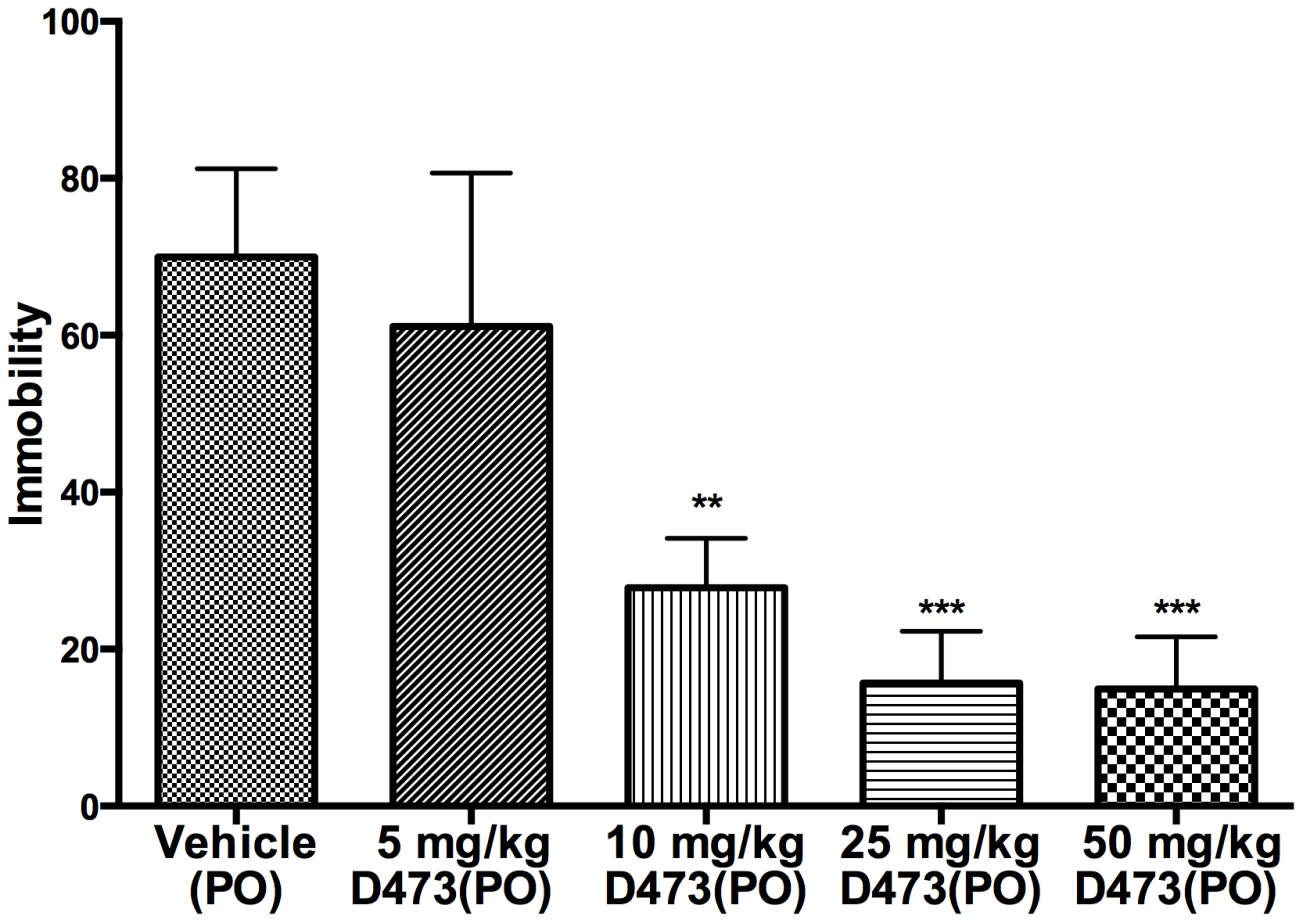
Effect of sub-chronic oral administration of vehicle and D-473 on the duration of immobility in the forced swimming test in rats. One way ANOVA analysis demonstrates significant effect among treatments: F (4, 24) = 10.33 (P<0.0001). Dunnett’s analysis showed that the effect of **D-473** at three doses (10, 25 and 50 mg/kg) on immobility was statistically significant different compared to vehicle (P<0.01).

D-473 was tested according to Prosolt’s forced swim procedure under oral administration conditions [30]. Significant effects in reduction of immobility compared to vehicle were observed at all three doses tested. The effect from the two highest doses (50 and 25 mg/kg) was comparable (Figure 4). The intermediate dose (10 mg/kg) also produced a robust, statistically significant effect. However, the lowest dose (5 mg/kg) did not produce significant reduction in immobility.

### In vivo data from locomotor activity test (oral administration)

**Figure 5:**
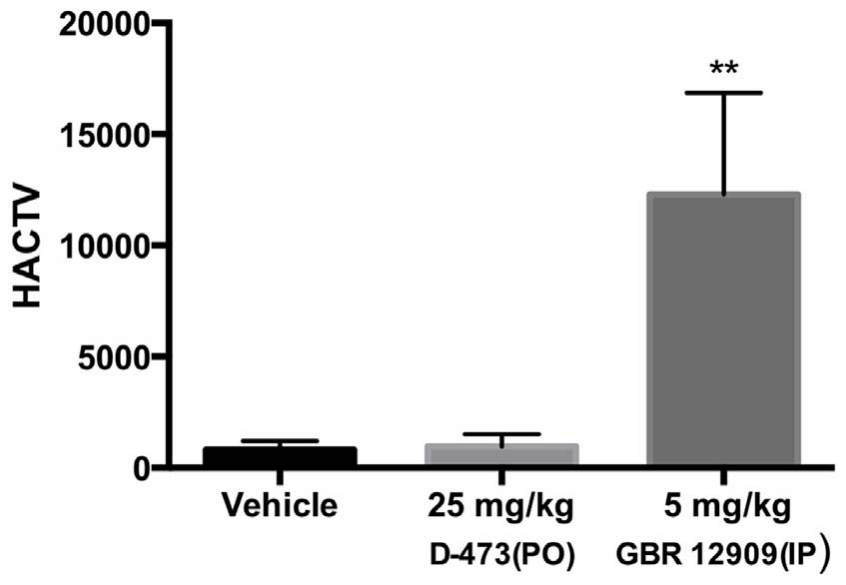
Effects of D-473 (PO) and GBR 12909 (i.p), on locomotor activity (horizontal activity, HACTV, measured by the number of infrared photobeam interuptions). D-473 was administered orally to the rats whereas Vehicle and GBR 12909 were injected i.p. The cumulative locomotor activities up to 90 min were measured. One way ANOVA analysis and subsequent Dunnett’s multiple comparisons testing demonstrates no significant difference between control and D-473 but a significant effect between control and GBR 12909: F (2,6) = 18.22 (P<0.0028). Asterisks indicate a statistically significant difference compared with Vehicle, **P<0.002. Each treatment group contains four to five rats.

An oral dose (25 mg/kg) of D-473 was tested for its effect on locomotor activity in rats and the result is shown in Figure 5. The activity from the drug measured for 2 h following post administration was statistically indistinguishable from vehicle. In contrast, this dose of D-473 was able to produce a significant effect in reducing immobility in the rat forced swim test (P<0.001, Figure 4). On the other hand, the classical DAT blocker GBR 12909 at a dose of 5 mg/kg (i.p.) produced a significant rise (P<0.001) in locomotor activity and thus exhibited a much more stimulant effect.

### Plasma concentration and brain uptake study

**Figure 6:**
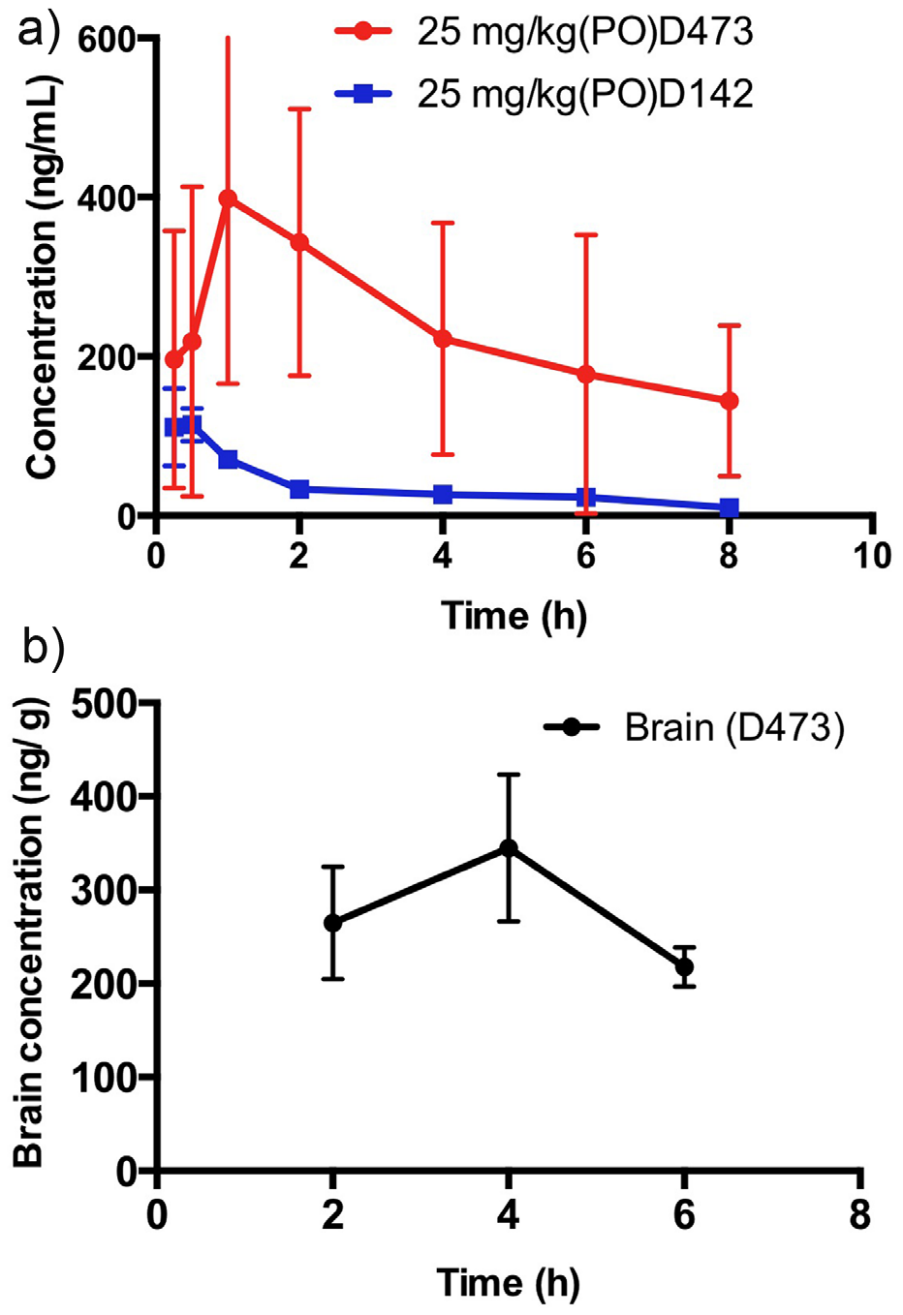
a) Average plasma concentration of D-473 and D-142 versus time following oral administration in male Sprague-Dawley rats at 25 mg/kg from a 3% HPßCD in water formulation. b) Time dependent concentration of D-473 in the brain following oral administration in male Sprague-Dawley rats at 25 mg/kg from a 3% HPßCD in water formulation.

Time dependent determination of plasma concentration of D-142 and D-473 was carried out over a period of 8 h upon oral administration of 25 mg/kg (PO) of each drug. Furthermore, the time dependent brain uptake of D-473 was also undertaken upon administration of 25 mg/kg (PO) of D-473 to measure penetration of D-473 into the brain. The drug concentration from brain and plasma was measured by LC/MS method as described above. D-473, following oral administration at 25 mg/kg, produced average maximum plasma concentration, Cmax of 404±223 ng/mL, reached between 1 and 2 hours (Figure 6a). The estimated average half-life value was found to be 6.39±0.106 hr. On the other hand, following oral administration of D-142 at 25 mg/kg, average Cmax was 125±36.6 ng/mL reached between15 and 30 minutes. The average half-life was found to be 2.50±0.96 hours (Figure 6a).

The brain uptake results indicate that the concentration of D-473 in the brain at 2 h was 264.5 ng/mL and the concentration peaked at 4 h (344.6 ng/mL) followed by a lowering of drug concentration at 6 h to values slightly lower than observed at 2 h. The results indicate a sustained level of brain drug concentration during many hours after oral administration (Figure 6b). The corresponding brain/plasma ratio ranged over values of 0.76, 1.54 and 1.22 at 2 h, 4 h and 6 h, respectively.

### Microdialysis in dorsolateral striatum and medial prefrontal cortex; changes in monoamines and metabolites

**Figure 7:**
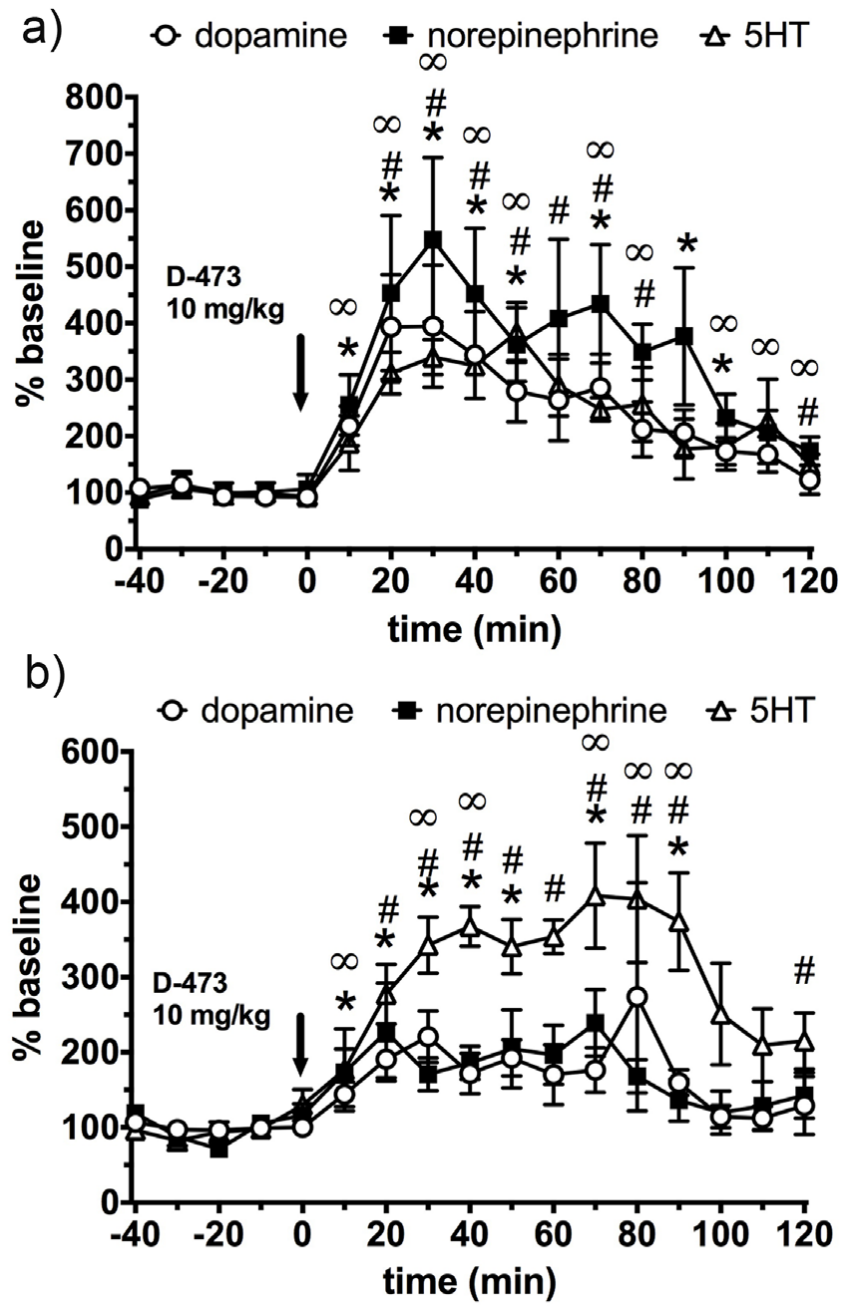
Time dependent effect of administration of D-473 (10 mg/kg, i.p) at time 0 (shown by arrow) on extracellular level of DA (Ο), 5-HT (▪), and NE (Δ); a) in rats Prefrontal cortex; and b) dorso lateral striatum. Results are expressed as percent baseline with baseline values all averaging 100%. Each point represents mean ± standard error (SE) of the percentage of baseline from five rats. Statistical analysis was performed by t-test analysis of every point relative to baseline values (i.e. 100%) using Prism 6 (Graphpad Software Inc, La Jolla, CA). * (DA) p<0.03-0.009; œ (NE) p<0.05- 0.01; # (5HT) p<0.05- 0.0001. P values <0.05 were considered to be statistically significant.

**Figure 8:**
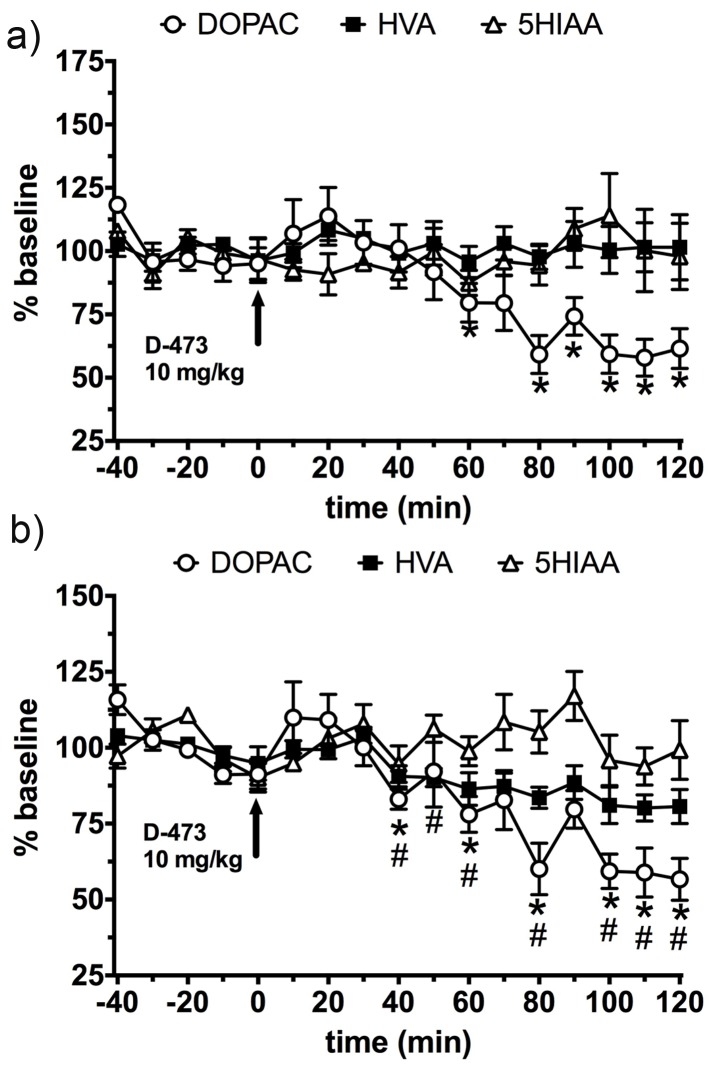
Time dependent effect of administration of D-473 (10 mg/kg, i.p) at time 0 (shown by arrow) on extracellular level of DOPAC (Ο), HVA (▪), and 5HIAA (Δ); a) in rats Prefrontal cortex; and b) Dorso lateral striatum. Results are expressed as percent baseline with baseline values all averaging 100%. Each point represents mean ± standard error (SE) of the percentage of baseline from five rats. Statistical analysis was performed by t-test analysis of every point relative to baseline values (i.e. 100%) using Prism 6 (Graphpad Software Inc, La Jolla, CA). * (DOPAC) p<0.02-0.0004. # (HVA) p<0.02–0.001. P values <0.05 were considered to be statistically significant.

Monoaminergic systems are essential regulators of movement, mood and behavioral states. In order to determine how the novel triple uptake inhibitor D-473 affects DA, NE, and 5-HT systems at the level of the prefrontal cortex (involved in cognitive states) and the dorsal lateral striatum (DLS), involved in motor activation, we systemically treated rats with D-473. A t-test was performed point to point from normalized basal levels (i.e. 100%) compared to post drug effects. D-473 significantly increased DA (maximal 394%), 5HT (maximal 339%), and NE (maximal 547%) in the mPFC (Figure 7a). At the end of two hours DA, 5-HT and NE remained elevated at 122%, 150% and 173% compared to the basal level (100%) in the mPFC region. Statistical analysis revealed that D-473 also significantly elevated DA (maximal 274%), 5-HT (maximal 403%), and NE (maximal 238%) in the DLS (Figure 7b). At the end of two hours DA, 5-HT and NE remained elevated at 129%, 215% and 142% compared to the basal level (100%) in the DLS region. DOPAC was reduced in the mPFC (maximal 35–40%, Figure 8a) and in the DLS (maximal 35–40%, Figure 8b) by systemic D-473. HVA was reduced in the DLS (maximal 20%).

## Discussion

MDD is one of the common forms of mental illness, which is not currently treated effectively. In spite of presence of large number of antidepressants, the majority of people often do not find relief from depressive disorders. It is well recognized now that different subtype forms of MDD exist in depression and not all subtypes are treated effectively by the current antidepressants [33], [34]. Two principle subtypes are melancholic and atypical depression possibly involving putatively different neurobiological mechanisms [14], [33], [35]. Patients with melancholic symptoms experience loss of appetite and weight loss, insomnia and psychomotor agitation as well as less responsive to environmental stimuli whereas atypical depression is associated with an increased appetite, weight gain, fatigue, hypersomnia and psychomotor retardation [35], [36]. This may be due to the fact that monoamines are affected differently in atypical and melancholic subtypes depression. The current antidepressant SSRIs and SNRIs do not alter dopaminergic activity, and, thereby, fail to address the melancholic component of MDD. This might explain their limitations in treating the overall symptomology in MDD resulting in a significant number of people failing to respond satisfactorily. Furthermore, SSRIs and SNRIs may act to inhibit DA activity via activation of certain subtypes of serotonin and adrenergic receptors [1], [5]. This could potentially make certain residual symptoms related to melancholic depression worse early on during treatment. On the other hand, TRIs should alleviate melancholic symptoms or anhedonia in addition to addressing atypical symptoms.

In this report, we show that D-473 exhibits a TRI-like profile at cloned human biogenic amine transporters expressed in HEK-293 cells. The relatively higher inhibitory potency at SERT compared with NET and DAT may be useful for production of beneficial antidepressant activity. It could be considered that potency at NET and DAT helps in reducing dependency on serotonin, and thus, improving the side effect profile. Among the three biogenic amine transporters, DAT showed the weakest interaction with D-473, suggesting a lowered risk for this compound to exhibit addiction liability. In this context, it can be noted that interaction with DAT, by itself, does not necessarily generate compounds with addictive potential [37]. Thus, some DAT inhibitors are known to lack stimulant properties, attributed to a specific preference for inward-facing conformations of DAT, or interaction with additional targets such as SERT, the cannabinoid CB1 receptor, or sigma receptors [37], [38], [39]. Moreover, another important factor besides inhibitory activity at DAT contribute to addictive potential: rapid central nervous system penetration [40].

One of our goals is to improve pharmacokinetic properties of TRI so that the drugs can be absorbed orally and cross the BBB. In order to increase in vivo stability of our lead TRI, we have introduced two fluorine atoms in the bi-phenyl rings of D-142. We found earlier that the compound D-142 posses poor oral bioavailability. As shown in Figure 6a, under oral administration conditions a dose of 25 mg/kg of D-473 exhibited a far superior profile with higher plasma concentration of the drug as well as longer half-life compared to D-142. In regards to brain uptake of D-473, as shown in Figure 6b the compound exhibited significant penetration into the brain. Interestingly, brain uptake of D-473 peaked at a later time point compared to the time point for peak plasma concentration. The brain uptake results (Figure 6b) indicate a persistence of D-473 in the brain which is also consonant with findings in in vivo experiments including microdialysis results. These findings indicate that introduction of fluorine atoms significantly improves in vivo stability of D-473.

An in vivo Forced Swim test (FST) with D-473 was carried out following oral administration of the compound. The drug produced a dose-dependent reduction of immobility with the two highest doses (50 and 25 mg/kg) producing comparable, maximal efficacy. The lower dose of 10 mg/kg still produced a robust, statistically significant reduction compared to vehicle (Figure 4). Overall, the results indicate that the drug was efficacious in reducing immobility. In order to assess whether D-473 was capable, by itself, to increase locomotion and thereby affect FST results, we evaluated locomotor activity of the drug at a dose that was active in the FST. Therefore, locomotor activity was monitored after oral administration of 25 mg/kg of D-473. As shown in Figure 5, locomotor activity was not significantly different from that observed with vehicle. Since we have shown that non-specific interactions between D-473 and a wide array of important CNS receptors are little to none, it is reasonable to conclude that pharmacological efficacy in the FST derives from the interaction of the drug with monoamine transporters.

In vitro uptake inhibition of three monoamine transporters by D-473 was reflected in the results obtained in the microdialysis study. The microdialysis data indicated that systemic administration of D-473 elevated all three monoamines significantly over baseline. This is in consistent with blockade of monoamine transporters in vivo by D-473 resulting in elevation of DA, 5-HT, and NE. D-473 robustly increased 5-HT (>3-fold), DA (4-fold) and NE (>5-fold) in the mPFC (Figure 7). Thus, in the prefrontal cortex we observed the highest elevation of NE followed by DA and 5-HT. In comparing these elevations of monoamine with in vitro action of D-473 on monoamine uptake one should take into account two issues. First, in prefrontal cortex, where levels of DAT are minimal compared with NET, uptake of DA is achieved mainly by NET [41]; thus, effects of an inhibitor on DA reflect mostly its interaction with NET. Second, the microdialysis effects are expressed as % baseline, and therefore baseline levels (which reflect the sum total of amine release and amine uptake) are factored in into the drug effect. Be that as it may, the elevation of serotonin in addition to that of DA and NE is important, because a monoamine imbalance has been observed in the mPFC area in depression, and therefore restoration of activity of all three monoamines should have an impact in relieving depressive symptoms [6], [7].

Overall, the profile of elevation of monoamines for D-473 in the mPFC area is similar to that for other reported TRIs although there are some interesting differences as well [16], [42], [43]. Most notably, the extent of elevation of NE with D-473 appears to be higher than reported with other TRIs. In contrast, for dorsolateral striatum there is no information regarding the effect of TRIs on dialysate monoamines. Dopaminergic activity in this region has been implicated in drug effects on motivation, learning, and cognition [44], [45]. Interestingly, in the DLS brain area, we find that the elevation of 5-HT over the basal level by D-473 was the highest (>4-fold) followed by the increases in DA and NE (2-fold) (Figure 7). Such increase in SERT activity in the DLS area seems to correspond to in vitro data shown in Table 1. Our previous microdialysis studies in the ventral tegmental area [46] and nucleus accumbens [47] showed a positive association between extracellular NE and DA output, rather than between 5-HT and DA output; it is therefore important for a TRI to interact with DAT as well as SERT (or NET), as elevations of endogenous 5-HT will not facilitate DA release contrary to literature on exogenous 5-HT agonists would suggest [46]. The reductions (up to 35%) observed in dialysate DOPAC with D-473 (Figure 8) are in line with previous reports on DAT inhibitors, generally describing little or moderate decreases depending on the DA terminal region studied [48], [49] as opposed to major reductions in DOPAC (∼65%) seen with a DAT releaser such as amphetamine [50]. The present results indicate reduction of HVA in the DLS but a lack of effect in the mPFC (Figure 8). DAT inhibitors have been reported to decrease or not change dialysate HVA levels in dopamine terminal regions [48], [49], [50]. We did not observe any change in the level of 5-HIAA. As we probe more into the neurochemical mechanism of this compound in the future, we hope to achieve a better understanding of the 5-HIAA results.

There has been a renewed interest in developing antidepressants with efficacy greater than currently existing antidepressant drugs as there is a significant unmet need in the current treatment of depression. Dopamine has been shown to play a significant role in depression. A dopaminergic drug like bupropion has been shown to be an effective antidepressant in the clinical setting. The hypothesis behind developing drugs acting at all three monoamine transporters as efficacious antidepressants has led to development of a number of TRIs. However, while all these drugs are referred to as TRIs, their capability of occupying the three monoamine transporters in vivo may not be similar. It is hypothesized that an optimal transporter occupancy is important for good efficacy. The concern about abuse liability of TRIs due to increased dopaminergic activity has been alleviated in recent studies demonstrating no such liability [16], [20].

In this work we describe the development of a novel TRI, D-473, an inhibitor of monoamine uptake at all three biogenic amine transporters with preference for SERT (Table 1). Furthermore, in an extended CNS receptor screening study, D-473 exhibited little to no affinity for other important CNS receptors. This compound showed higher in vivo stability and oral activity compared to our previous closely related analog D-142. The drug D-473 exhibited good brain penetration and produced efficacious activity in an animal model of depression. The optimal efficacy dose in the swim test did not produce any locomotor activation. Microdialysis experiment demonstrated that systemic administration of D-473 elevates extracellular level of the three monoamines DA, 5-HT, and NE, indicating in vivo blockade of all three monoamine transporters by D-473.

## Supporting Information Legends

Figure S1 **Placement of microdialysis probes.** Representative implantation of microdialysis probes in the dorsal lateral striatum and medial prefrontal cortex. Images of the brain slices are taken from Paxinos and Watson, 2007.
(TIF)
