# Supplementary figures and images for: Pharmacological and Behavioral Characterization of D-473, an Orally Active Triple Reuptake Inhibitor Targeting Dopamine, Serotonin and Norepinephrine Transporters
Source: PLoS One. 2014 Nov 26;9(11):e113420. doi: 10.1371/journal.pone.0113420 (PMC4245125; doi:10.1371/journal.pone.0113420)

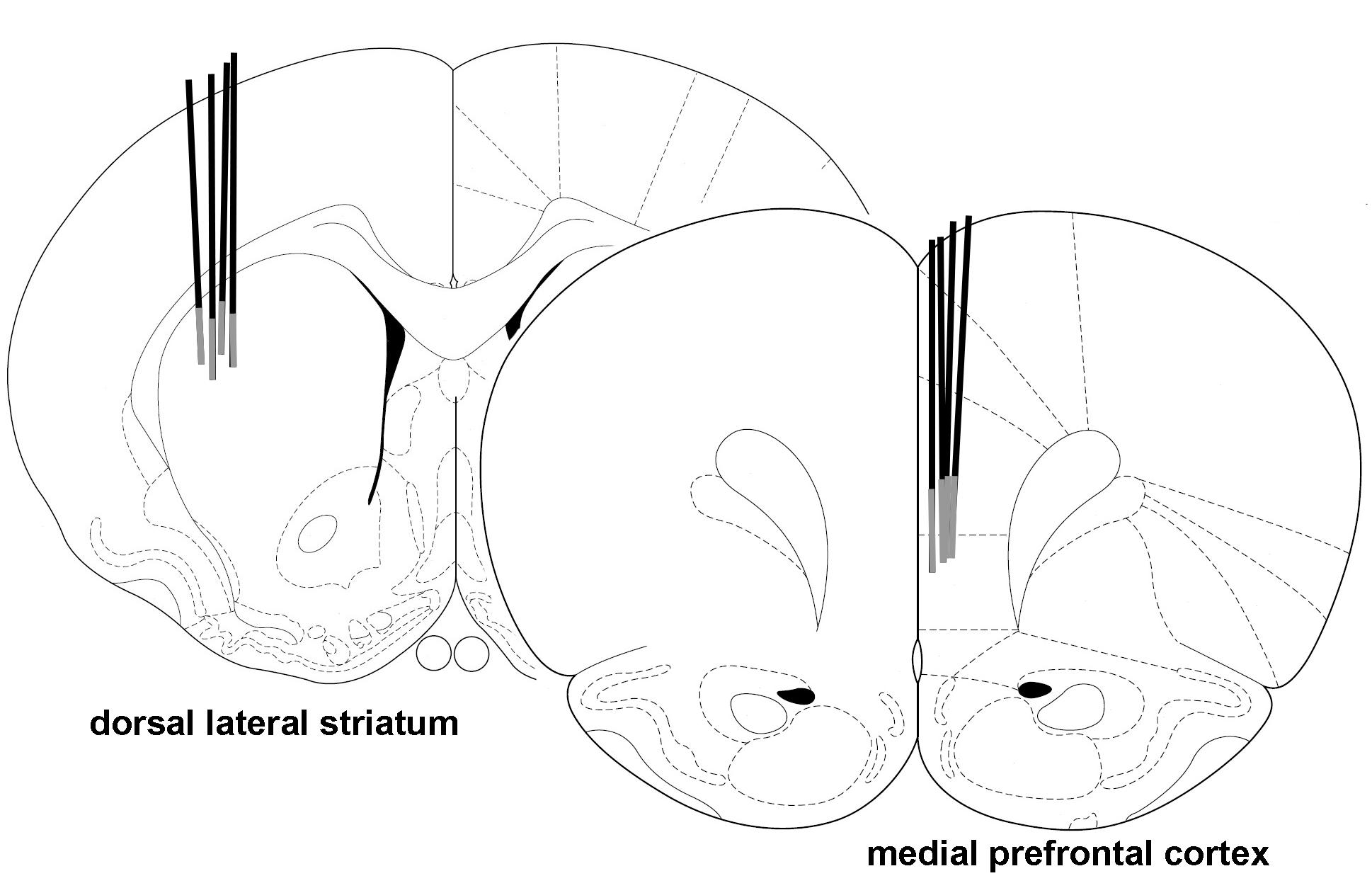

Supplement: Figure S1 — Placement of microdialysis probes. Representative implantation of microdialysis probes in the dorsal lateral striatum and medial prefrontal cortex. Images of the brain slices are taken from Paxinos and Watson, 2007. (TIF) [file pone.0113420.s001.tif]
